# Beta-hydroxybutyrate (3-OHB) can influence the energetic phenotype of breast cancer cells, but does not impact their proliferation and the response to chemotherapy or radiation

**DOI:** 10.1186/s40170-018-0180-9

**Published:** 2018-06-11

**Authors:** Catharina Bartmann, Sudha R. Janaki Raman, Jessica Flöter, Almut Schulze, Katrin Bahlke, Jana Willingstorfer, Maria Strunz, Achim Wöckel, Rainer J. Klement, Michaela Kapp, Cholpon S. Djuzenova, Christoph Otto, Ulrike Kämmerer

**Affiliations:** 1 Department of Obstetrics and Gynaecology, University Hospital of Würzburg, Josef-Schneider-Str. 4, 97080 Würzburg, Germany; 2 Department of Biochemistry and Molecular Biology, Theodor-Boveri-Institute, Biocenter, University of Würzburg, 97070 Würzburg, Germany; 3 Department of Radiotherapy and Radiation Oncology, Leopoldina Hospital, 97422 Schweinfurt, Germany; 4 Department of Radiotherapy, University Hospital of Würzburg, 97080 Würzburg, Germany; 5 Experimental Surgery, Department of General, Visceral, Vascular, and Pediatric Surgery, University Hospital of Würzburg, 97080 Würzburg, Germany

**Keywords:** Ketogenic diet, β-Hydroxybutyrate, Ketone bodies, Breast cancer, Seahorse, Metabolic profile, Chemotherapy, Ionizing radiation

## Abstract

**Background:**

Ketogenic diets (KDs) or short-term fasting are popular trends amongst supportive approaches for cancer patients. Beta-hydroxybutyrate (3-OHB) is the main physiological ketone body, whose concentration can reach plasma levels of 2–6 mM during KDs or fasting. The impact of 3-OHB on the biology of tumor cells described so far is contradictory. Therefore, we investigated the effect of a physiological concentration of 3 mM 3-OHB on metabolism, proliferation, and viability of breast cancer (BC) cells in vitro.

**Methods:**

Seven different human BC cell lines (BT20, BT474, HBL100, MCF-7, MDA-MB 231, MDA-MB 468, and T47D) were cultured in medium with 5 mM glucose in the presence of 3 mM 3-OHB at mild hypoxia (5% oxygen) or normoxia (21% oxygen). Metabolic profiling was performed by quantification of the turnover of glucose, lactate, and 3-OHB and by Seahorse metabolic flux analysis. Expression of key enzymes of ketolysis as well as the main monocarboxylic acid transporter MCT2 and the glucose-transporter GLUT1 was analyzed by RT-qPCR and Western blotting. The effect of 3-OHB on short- and long-term cell proliferation as well as chemo- and radiosensitivity were also analyzed.

**Results:**

3-OHB significantly changed the oxygen consumption rate (OCR) and extracellular acidification rate (ECAR) in BT20 cells resulting in a more oxidative energetic phenotype. MCF-7 and MDA-MB 468 cells had increased ECAR only in response to 3-OHB, while the other three cell types remained uninfluenced. All cells expressed MCT2 and GLUT1, thus being able to uptake the metabolites. The consumption of 3-OHB was not strongly linked to mRNA overexpression of key enzymes of ketolysis and did not correlate with lactate production and glucose consumption. Neither 3-OHB nor acetoacetate did interfere with proliferation. Further, 3-OHB incubation did not modify the response of the tested BC cell lines to chemotherapy or radiation.

**Conclusions:**

We found that a physiological level of 3-OHB can change the energetic profile of some BC cell lines. However, 3-OHB failed to influence different biologic processes in these cells, e.g., cell proliferation and the response to common breast cancer chemotherapy and radiotherapy. Thus, we have no evidence that 3-OHB generally influences the biology of breast cancer cells in vitro.

**Electronic supplementary material:**

The online version of this article (10.1186/s40170-018-0180-9) contains supplementary material, which is available to authorized users.

## Background

Breast cancer (BC) is one of the most common cancers and affects about one in eight women during their lifetime [1]. In general, modern BC therapy includes different therapeutic approaches, such as surgical removal of the tumor, chemotherapy, radiation, and hormone therapy [2]. In addition to these conventional therapies, a large number of patients seek supportive therapies like specific diets to improve their outcome. The correlation between different types of diet and the incidence and progression of cancer is increasingly becoming the focus of research [3–7]. In this respect, avoiding carbohydrates to specifically “starve cancer cells” is the most popular trend amongst “cancer diets.” The rationale for this dietary regime is often based on the “Warburg effect,” which describes the preferential fermentation of glucose to lactate even under availability of sufficient oxygen [8, 9]. Therefore, reducing carbohydrate intake and thus lowering blood glucose seems to be a promising strategy for cutting cancer off from glucose supply [10–12].

Besides fasting, the strictest form of such a “very low carb” diet is called the ketogenic diet (KD). The KD is characterized by consuming the predominant proportion of calories from fat, balancing those derived from protein and thus consuming very few calories from glucose or other carbohydrates. Different KD regimens were shown to be safe and well tolerated in a variety of malignancies [13–19] and lead to the metabolic state of a physiological ketosis [20]. During ketosis, the “ketone bodies” acetoacetate (AcAc) and D-β-hydroxybutyrate (R-3-hydroxybutyrate: 3-OHB) are predominantly produced in the liver and can be detected in the peripheral blood and urine above normal levels [21]. 3-OHB is found at similar or higher concentrations than AcAc and therefore, considered the principal “ketone body.” In humans, the median concentration of 3-OHB in plasma reaches approximately 3 mM under short-term fasting conditions [22], up to 6 mM during long-term starvation [23] and regularly at least 2 mM under a ketogenic diet [24].

3-OHB is transported into cells via monocarboxylic acid transporters (MCT). The isoforms MCT1, MCT2, and MCT4 can transport lactate and ketone bodies across the cell membrane [25, 26]. Here, MCT2 has the highest affinity for 3-OHB, whereas MCT1 and 4 have a higher affinity for lactate [27, 28]. In mitochondria, 3-OHB is degraded via ketolysis into acetyl-CoA, which then is metabolized within the Krebs cycle and the respiratory chain to generate energy [29–32]. Since the oxidation of 3-OHB generates more energy per mol oxygen used compared to glucose, it is sometimes labeled a “superfuel” [33]. However, cells need functioning mitochondria as well as sufficient oxygen supply to generate energy from 3-OHB. The latter is hampered in the hypoxic microenvironment of larger tumors, which has already been shown in vivo for breast cancer tissue in patients [34].

There are somewhat contradicting results regarding the effect of 3-OHB on growth and biology of tumor cells cultured in vitro and in experimental tumors in mice. In some studies, ketone bodies seem to be associated with cancer progression, metastasis, and poor clinical outcome [35, 36]. In contrast, it was shown that a ketogenic diet significantly reduces tumor growth in mice [37, 38]. Further, an antiproliferative effect of 3-OHB was already shown for different cancer cells, such as glioblastoma and tumor stem cells [37], melanoma, cervical carcinoma, or neuroblastoma [39–41]. Several studies also described a significant delay of tumor growth in mice and humans in a systemic ketosis [16, 18, 37, 38, 40, 42–49]. In this respect, Rodrigues and coworkers reported evidence for a “β-hydroxybutyrate paradox” [50]. They postulated that the effect of 3-OHB on cancer growth would depend on the tumor’s energetic phenotype. Thus, “oxidative cells” would use 3-OHB as an additional energy source so that tumors with predominantly “oxidative cells” increase their growth when this metabolite is available. Other cells with a more “glycolytic, Warburg-like phenotype” would be unable to metabolize 3-OHB in which case it could accumulate intracellularly and inhibit tumor growth via signaling and epigenetic mechanisms [50].

In view of this preclinical pro- and contra evidence and the fact that increasing numbers of patients are adopting a ketogenic diet or short-term fasting during oncological therapy, we studied the impact of 3-OHB on seven different BC cell lines in vitro. Here, we initially analyzed the energetic profile of these cells and correlated this to the effect of 3-OHB on cell proliferation. Further, we investigated the possibility of synergism between ketosis and radio- or chemotherapy [51]. To mimic a physiological state of metabolites found in the circulation of patients performing a KD, we performed experiments with 3 mM 3-OHB, representing pronounced ketosis, and 5 mM glucose, typical for the blood glucose level found in persons on a KD (range 4.9–5.2 mM) [19, 52–57]. Furthermore, we investigated cells at both an oxygen supply of 5% oxygen (= 5 kPA), a typical mean concentration between well-vascularized benign breast tissue (6.5 kPA) and non-hypoxic tumor regions (3.2–4.7 kPA) in vivo [58] and 21% oxygen (21 kPA), as a common condition used in cell culture.

## Methods

### Breast cancer cell lines and culture

The BC cell lines BT20, BT474, HBL100, MCF-7, MDA-MB 231, MDA-MB 468, and T47D were obtained from Cell Lines Service GmbH (CLS; Eppelheim, Germany), and their receptor status, subtype, and mutation status are summarized in Table 1 [59–65]. The BT474 and the MDA-MB 231 cell lines were purchased directly from CLS for the experiments and used at low passage. All other cell lines were authenticated via genetic profiling of SRT loci by CLS before running the experiments. Aliquots of the cell lines were freshly cultured from frozen samples in 75-cm^2^ cell culture flasks (TPP, Trasadingen, Switzerland) in Dulbecco’s modified Eagle’s medium (DMEM)/Hams F12 (1:1) medium (Gibco, ThermoFisher Scientific, Darmstadt, Germany) supplemented with 10% fetal calf serum (FCS; Biochrom, Berlin, Germany) and 50 ng/ml Gentamycin (Sigma-Aldrich, Munich, Germany) in the presence of 5% CO_2_ and 21% oxygen, respectively.

**Table 1:** Subtype of the breast cancer cell lines used in the experiments

Cell line	Receptor			Subtype	Ref.	Mutations*
	ER	PR	Her2			
BT20	−	(−)	np	Basal A	[60]	ATM; BRCA2; CBLB; CDKN2A; COL1A1; RAP1GDS1; RB1; PIK3CA TP53
	0	0	0–1	Basal	[61]	
BT474	+	(+)	+	Luminal	[60]	EPS15; HIST1H3B; NSD1; PIK3CA; PPP2R1A; RHOA; TP53
	0	8	3+	Luminal B	[61]	
	+	+/−	+	Luminal B	[59]	
HBL100	−	(−)	np	Basal B	[60]	np
MCF-7	+	(+)	np	Luminal	[60]	ATP2B3; CDKN2A; EP300; ERBB4; MAP3K13; PIK3CA
	6	6	0–1	Luminal A	[61]	
	+	+/−	−	Luminal A	[59]	
MDA-MB 231	−	(−)	np	Basal B	[60]	BRAF; CD79A; KRAS; CNKN2A; NF2; PBRM1; PDGFRA; TP53
	0	0	0–1+	Basal	[61]	
MDA-MB 468	(−)	(−)	np	Basal A	[60]	CACNA1D; INPP4B; PTEN; RB1; TP53
	0	0	0	Basal	[61]	
	−	−	−	Basal	[59]	
T47D	+	(+)	np	Luminal	[60]	ACVR1; ARID1A; PIK3CA; TP53
	+	+	−	Luminal A	[59]	

The subtype of the breast cancer cell lines used was classified before by gene expression profile and the expression of the estrogen (ER), progesterone (PR), and human epidermal growth factor receptor 2 (Her2) receptor. The abbreviations of receptors and breast cancer subtype classification shown here are published [59–61]. Semiquantified receptor status: results with “−”, “(−)”, “0”, and “0–1+” are classified as negative and results with “+”, “(+)”, “+/−”, 6, and 8 are classified as positive for receptor expression (also summarized by [62])

*np* not published

*Mutations shown here are described by [63–65]

### Turnover of metabolites

For quantification of glucose, lactate, and 3-OHB metabolism, cells were seeded and cultured at conditions described in the cell proliferation assay. After 5 days, supernatants were collected and the levels of 3-OHB were analyzed by the PrecisionXceed® instrument with the corresponding test strips FreeStyle Precision® β-Ketone (Abbott, Wiesbaden, Germany). The concentrations of glucose and lactate were measured with the Cobas 8000 modular analyzer series (Roche Diagnostics; Mannheim, Germany) at the central laboratory of the University Hospital of Würzburg. Concentrations of metabolites were expressed in millimolar per optical density (OD) of crystal violet dye extracts in each well at day 3 or 5 of culture. The amount of solubilized dye in OD is directly proportional to the cell number. Therefore, after removing the supernatant carefully for metabolite quantification, adherent cells were fixed with 100 μl methanol (Sigma-Aldrich) for 10 min at room temperature (RT) and then dried. Cells were stained by incubation in 100 μl crystal violet solution per well (0.4% crystal violet [Merck, Darmstadt, Germany] in 1:3 methanol: phosphate-buffered saline) for 10 min at RT and then washed several times with distilled water. Crystal violet was extracted from cells with 100 μl of 10% acetic acid per well on a plate shaker for 30 min, and OD was determined at 570 nm by using a standard ELISA-Plate reader.

### Energetic profiling by Seahorse technique

The oxygen consumption rate (OCR) and extracellular acidification rate (ECAR) were analyzed with the Seahorse XF Cell Mito Stress Test (Part #103015-100; Agilent Technologies, Santa Clara, CA, USA) in a Seahorse XFe96 Analyzer (Agilent Technologies). The day before the experiment, 40,000 cells per well were plated in a 96-well Seahorse plate in 100 μl DMEM/10% FCS/Gentamycin/5 mM glucose medium with or without 3 mM 3-OHB (sodium-hydroxybutyrate, Sigma-Aldrich). The Agilent Seahorse XFe96 Sensor Cartridge was hydrated with 200 μl/well of XF calibrant solution overnight in a non-CO_2_ incubator at 37 °C. On the day of the experiment, 100 ml of Seahorse assay medium containing 1 mM pyruvate, 2 mM glutamine, and 5 mM glucose was prepared. The pH of the pre-warmed (37 °C) medium was adjusted to 7.4 with 0.1 N NaOH. Twenty milliliters of the assay medium was used to prepare 3 mM 3-OHB, and the pH was readjusted to 7.4 with 0.1 N HCl. Cells were washed twice with 200 μl of the corresponding Seahorse medium and incubated in 175 μl of the respective Seahorse medium per well in a non-CO_2_ incubator at 37 °C for 1 h. Meanwhile, the Seahorse sensor cartridge ports were loaded with 25 μl of inhibitors to have a final concentration of 2 μM oligomycin (port A, Calbiochem), 1 μM FCCP (port B, Sigma-Aldrich), and 0.5 μM rotenone/antimycin A (port C, Sigma-Aldrich). The experimental design was setup using the WAVE software program, and measurements were performed in the Seahorse XFe96 Analyzer. After the measurement, supernatant from the cells was removed and the cells were fixed by addition of 100 μl methanol (Sigma-Aldrich) for 10 min at RT and air dried. Subsequently, the cells were stained using crystal violet solution as described for the colony formation assay (see below). For quantification, stained plates were incubated with 200 μl of 10% acetic acid per well with shaking for 15 min and the resulting solution was analyzed in a plate reader (Tecan GENios plus, Tecan Deutschland GmbH, Crailsheim, Germany) at 630 nm.

### Cell proliferation assay

Adherent growing cells were seeded in 96-well flat bottom plates (TPP) at cell numbers determined for each cell line to reach semiconfluency after 3 days under the respective oxygen and low glucose conditions and to reach confluency after 5 days via preliminary testing. Thus, 250–350 cells per well, depending on the cell line, were seeded in 200 μl DMEM/10%FCS/Gentamycin/5 mM glucose. Cell plates were cultured for 5 days at 5% CO_2_ and 37 °C in humidified chambers at oxygen concentrations of 21 or 5% in hypoxia-incubators (Coy Laboratories Products Inc., Grass Lake, MI, USA) respectively. At least 3 independent wells per cell line were tested either with or without 3 mM 3-OHB or 1.5 mM acetoacetate (lithium salt) and LiCl (both Sigma-Aldrich) as control in parallel. Four independent experiments were performed with fresh cultured cell aliquots. At day 5, supernatants were removed for metabolite testing, 100 μl of fresh medium containing 5-bromo-2′-deoxyuridine (BrdU) were added for another 24 h, and cell proliferation rate was then analyzed by the BrdU test (Roche; Cell Proliferation ELISA) according to the manufacturer’s instructions.

### Colony formation assay

For the colony formation assay, cells were cultured at 100 cells/well in 48 well plates (TTP) with 500 μl medium (DMEM/10% FCS/Gentamycin/5 mM glucose) with or without 3 mM 3-OHB at oxygen concentrations of 5 or 21% for 14 to 18 days, depending on the cell line. Half of the medium was replenished every 4 days. Crystal violet staining was performed for evaluation of the adherent colonies. Therefore, supernatant was carefully removed; adherent cells were fixed with 250 μl methanol (Sigma-Aldrich) for 10 min at RT and then dried. Colonies were stained by incubation in crystal violet solution (same as above) for 10 min and then washed several times with distilled water. Stained colonies were documented by using the ImmunoCapture 6.2 in the ImmunoSpot Analyzer® (Cellular Technology, Shaker Heights, OH, USA). Two independent experiments were performed with 8 replicate wells per condition (5 and 21% oxygen with and without 3-OHB) for each cell line.

### Reverse transcriptase quantitative polymerase chain reaction (RT-qPCR) and Western blotting

For RT-qPCR and Western blot analyses, aliquots of cells were cultured under the same medium (DMEM/10% FCS/Gentamycin/5 mM glucose; with or without 3 mM 3-OHB) and oxygen (21 or 5%) conditions to reach subconfluency after 5 days in 75-cm^2^ cell culture flasks.

RNA extraction, cDNA synthesis (iScript, Bio-Rad), and qPCR (Mesa Green containing Meteor Taq hotstart polymerase, Eurogentic) as well as Western blotting were performed as described previously [66]. The primers and conditions of the qPCR are described in Table 2. In brief, qPCR reactions were performed on a CFX96 real-time PCR system (Bio-Rad) operated by CFX Manager Software (version 3.0). The cycler protocol was 5 min at 95 °C (initial denaturation), 40 cycles of 15 s at 95 °C, 60 s at 60 °C (two-step protocol), and 5 min at 72 °C (final extension) for all primer pairs used. Fold expression of genes of interest (Table 2) expression relative to reference genes PPIA and β-actin was calculated with the ∆∆Cq method [67]. Post-amplification melting curves were controlled to exclude primer-dimer artifacts and contaminations (not shown). Data from two independent cell culture experiments in triplicate reactions for each primer pair were summarized (Fig. 3b).

**Table 2:** Details of the primer pairs used in this study

Gene	Forward primer/reverse primer	cDNA size (bp)	PubMed access no.
β-Actin	5′-CCT TGC CAT CCT AAA AGC C-3′ 5′-CAC GAA AGC AAT GCT ATC AC-3′	96	NM_001101
PPIA	5′-TGT CCA TGG CAA ATG CTG GAC CC-3′ 5′-GCG CTC CAT GGC CTC CAC AA-3′	140	NM_021130.3
BDH1	5′-CGC CGG GTG AAG GCG-3′ 5′-GAA TGG CCC AGT TCC TCC C-3′	92	NM_004051.4
SCOT	5′-GCC ATT GCC AGT AAG CCA AG-3′ 5′-CCA GGC TTT CAC CAA AGC AA-3′	99	NM_000436.3
ACAT1	5′-CGG CAG ATG CAG CGA AGA GG-3′ 5′-AGG TTC TAC AGC AGC GTC AGC A-3′	77	NM_000019.3

DNA sequences and PubMed accession numbers for each gene are indicated. qPCR reactions were performed on a CFX96 real-time PCR system (Bio-Rad) operated by CFX Manager Software (version 3.0). The two-step cycler protocol was 5 min at 95 °C, 40 cycles of 15 s at 95 °C, 60 s at 60 °C, followed by 5 min at 72 °C and used for all primer pairs

*PPIA* peptidylprolyl isomerase A, *BDH* 3-hydroxybutyrate dehydrogenase, *SCOT* succinyl-CoA transferase (3-oxoacid CoA transferase), *ACAT* acetyl-CoA-acetyltransferase

For Western blotting, pellets of 1 × 10^6^ tumor cells were lysed and protein concentrations were determined using the Bradford method [68] and Coomassie Brilliant Blue (Roti-Quant; Roth, Karlsruhe, Germany) reagent. Afterwards, samples were mixed with 5× loading buffer (Fermentas GmbH, St. Leon-Roth, Germany), denatured at 95 °C for 5 min, chilled on ice, and stored at − 20 °C for further analysis. Equal amounts of proteins (20 μg) were separated on a 10% polyacrylamide gel. The antibodies used are listed in Table 3.

**Table 3:** Antibodies for Western blot

Antibody	Gen/name	Company	Clone	Species	Dilution	MW of antigen
Primary	Beta-actin	Abcam	mAbcam 8226	Mouse	1:10000	42 kDa
	Monocarboxylic acid transporter 2 (MCT-2)	Abcam	Polyclonal	Rabbit	1:2000	52 kDa
	Glucose transporter 1 (GLUT1)	USBiological	Polyclonal	Rabbit	1:1000	55 kDa
Secondary	HRP-labeled goat-anti-mouse	KPL	Polyclonal	Goat	1:10000	
	HRP-labeled goat-anti-rabbit	Abcam	Polyclonal	Goat	1:20000	

*MW* molecular weight

### Chemosensitivity and radiosensitivity

For both sensitivity tests, the cells were seeded as in the prior tests in 96-well plates in standard medium with 5 mM glucose and incubated for 48 h at oxygen concentrations of 5 or 21% respectively.

For chemosensitivity testing, the test drug concentration (TDC) of epirubicin was defined as 0.5 μg/ml, of paclitaxel as 13.6 μg/ml, and of carboplatin as 15.8 μg/ml, as described in the literature [69–72]. The cell culture medium (see above) with or without 3 mM 3-OHB and eight different dilutions of the chemotherapeutic drugs (epirubicin: highest concentration: 25% TDC and twofold dilution series; paclitaxel highest concentration: 50% TDC and tenfold dilution series or carboplatin highest concentration: 400% TDC and twofold dilution series) were added. TDC concentrations for the experiments were selected by cell viability assays performed in preliminary tests with all cell lines (not shown). After 3 days of cell culture, BrdU was added for the final 24 h of culture and the BrdU test performed as described above.

For radiosensitivity testing, subconfluent monolayers of cells were irradiated in the culture plates filled with 200 μl/well with the corresponding growth medium with graded single doses (0–8 Gy) and then cultivated for 10 days at either 5 or 21% oxygen concentration. Irradiation was performed using a 6 MV Siemens linear accelerator (Siemens, Concord, CA, USA) at a dose rate of 2 Gy/min. Half of the medium was changed every 4 days. At the end of the incubation period, BrdU was added and cell proliferation was measured by the BrdU test 24 h thereafter.

### Statistics

Data are presented as means (± standard error of mean [SEM]). *p* values lower than 0.05 in the non-parametric Mann-Whitney *U* test were considered significant. The software GraphPad Prism 6 (La Jolla, CA 92037 USA) was used to create the figures and to perform statistical analysis. IC50 was determined via the nonlinear regression dose-response curve (inhibition) function of the Prism software.

## Results

### Glucose consumption and lactate production is not influenced by 3-OHB

**Fig. 1:**
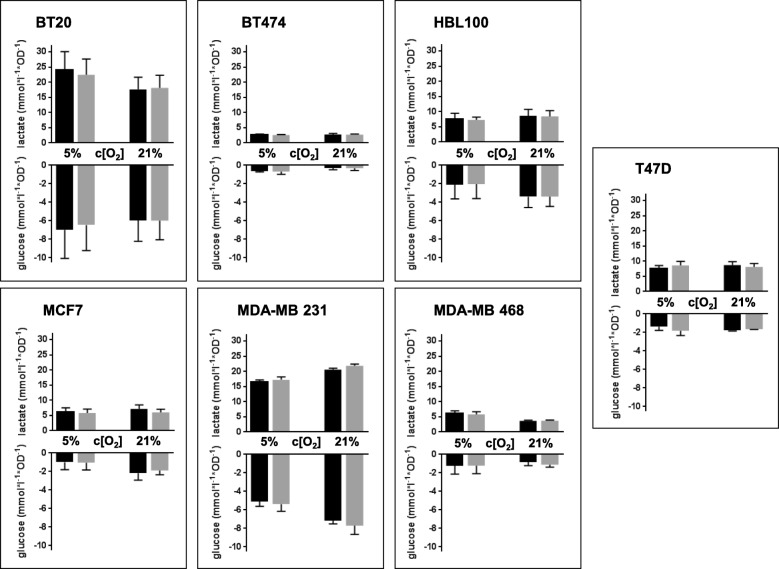
The graphs represent the rate of lactate production (mmol × l^−1^ × optical density (OD)^−1^; upper panel) and glucose consumption (mmol × l^−1^ × OD^−1^; lower panel) normalized to total cell content (OD) after 5 days of culture in 5 or 21% oxygen (gray column = 3 mM 3-OHB; black column = control). Each column represents mean ± SEM of four independent experiments

Glucose consumption and production of lactate were normalized to the optical density (OD) of crystal violet dye extracts, which is directly proportional to the cell number, as readout for the rate of aerobic glycolysis (Warburg effect) or respiration, respectively. With these measurements, we were able to categorize the metabolic characteristics of the seven tested cell lines as follows: BT20 and MDA-MB 231 exhibit high glucose consumption (with an average of more than 5.0 mM) normalized to cell number as reflected by OD readout of the crystal violet assay) and high lactate production (with an average of more than 15 mM/OD). HBL100, MCF-7, MDA-MB 468, and T47D consume relatively low amounts of glucose (< 3.5 mM/OD) and show low lactate production (< 10 mM/OD), while BT474 show very low levels of glucose consumption (< 1 mM/OD) and lactate production (< 5 mM/OD). Interestingly, independent of the respective metabolic characteristics, 3 mM 3-OHB failed to significantly influence the consumption of glucose or production of lactate in the presence of either 5 or 21% oxygen (Fig. 1).

### 3-OHB changes oxygen consumption rate and extracellular acidification rate in BT20 cells

**Fig. 2:**
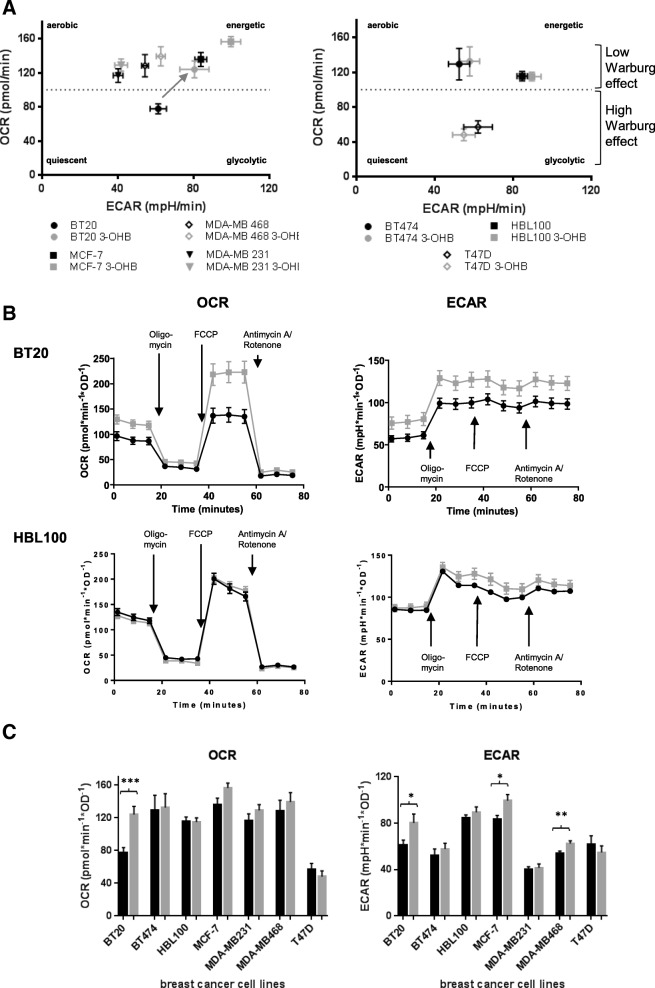
**a** Energetic phenotype as revealed by Seahorse flux analysis in cultures without 3-OHB (black symbols) and with 3 mM 3-OHB (gray symbols). Arrow indicates the significant (*p* < 0.05) shift in energetic phenotype observed with the BT20 cell line. Graph summarizes the results of four independent seahorse experiments with four replicate wells for each cell line. **b** The curves of OCR and ECAR for the BC cell lines with the most prominent changes (BT20) and without any changes (HBL100) depending on the addition of 3-OHB are shown here. The graph represents the three measuring points of basal levels of respiration/acidification, and changes after addition of oligomycin, FCCP, and antimycin A/rotenone (black line and dots = control, gray line and boxes = 3-OHB). **c** Column statistics of the baseline OCR and ECAR of BC cell lines with 3-OHB (gray column) compared to control (black column) (***p < 0.001; **p < 0.01; *p < 0.05). Each column summarizes mean ± SEM of four independent seahorse experiments with four replicate wells per experiment for each cell line

Seahorse analysis confirmed the metabolic phenotype found in the glucose consumption/lactate production rate analysis. All cell lines except T47D exhibited a more aerobic/energetic cell type, corresponding to cells that divide but generate their energy predominantly from oxidative phosphorylation (OXPHOS) (Fig. 2a).

This basal metabolic phenotype did not correlate with the reaction of these cells to 3-OHB, since 3-OHB significantly influenced the oxygen consumption rate (OCR) in BT20 cells only. Here, OCR increased from 77.8 ± 5.8 to 124.0 ± 10.0 pmol/min (mean ± SEM; *p* < 0.001), when cells were cultured in the presence of 3-OHB (Fig. 2b, c). For all other cell lines, we found no evidence that 3-OHB influenced oxygen consumption (Fig. 2c, left graph). In addition, the effect of 3-OHB on extracellular acidification rate (ECAR) was analyzed. In the case of BT20 cells, ECAR significantly increased from 61.2 ± 4.3 mpH (milli pH)/min to 80.3 ± 7.7 mpH/min (*p* < 0.05) with 3-OHB. An increase from 83.6 ± 3.1 mpH/min to 99.5 ± 5.2 mpH/min (*p* < 0.05) and from 54.2 ± 1.8 mpH/min to 62.5 ± 2.3 mpH/min (*p* < 0.01) was observed in MCF7 and MDA-MB 468 cells under the influence of 3-OHB, respectively (Fig. 2c, right side).

Thus in general, no direct correlation between the metabolic reaction to 3-OHB and the basal energetic phenotype of the cell lines was seen.

### Consumption of 3-OHB is not strongly linked to overexpression of ketolytic enzymes and does not correlate with the observed effects on metabolic phenotype

**Fig. 3:**
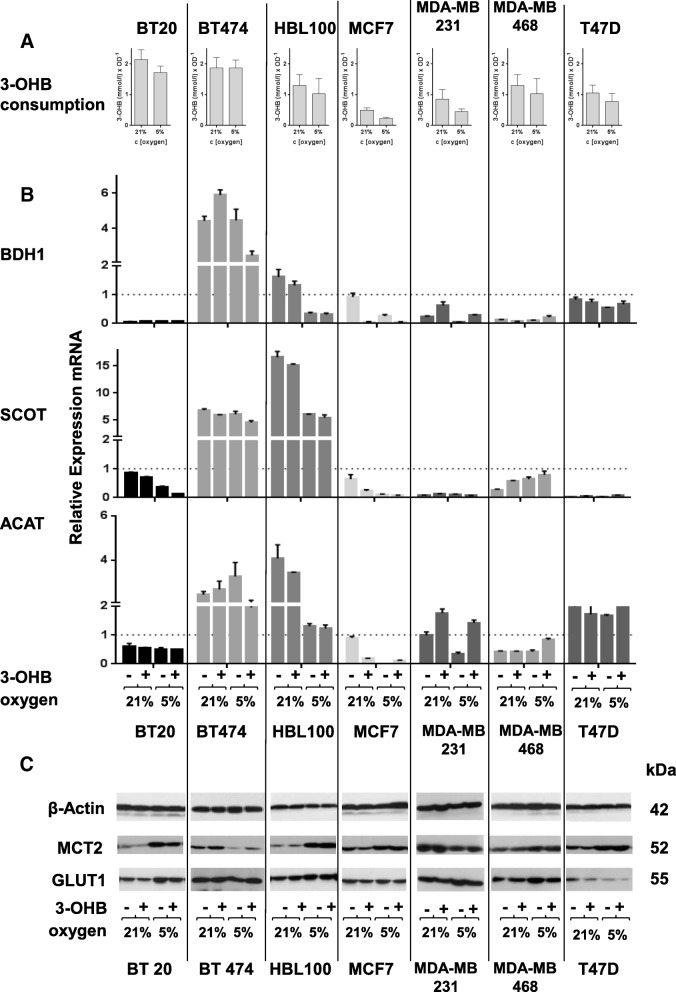
**a** The columns show the amount of 3-OHB (in mM) consumed by the cells normalized to their cell number as given by optical density (OD) measured with the crystal violet assay. Columns represent mean ± SEM of two independent experiments with three replicate wells per experiment. There was no significant difference in the consumption of 3-OHB between cultivation at 21 and 5% oxygen. A tendency to reduced 3-OHB consumption was observed at 5% oxygen. **b** Relative expression of mRNA for the ketolytic enzymes BDH1 (β-hydroxybutyrate dehydrogenase), SCOT (succinyl-CoA:3-ketoacid coenzyme A transferase), and ACAT (acetyl-CoA acetyltransferases) in the tested BC cell lines. Each column represents mean ± SEM of data from two independent cell culture experiments in triplicate reactions for each primer pair. **c** All cell lines express the most important transporter for 3-OHB, the monocarboxylate transporter 2 (MCT2), and the glucose transporter 1 (GLUT1) on protein level. Beta-actin served as loading control. Representative Western blot images of the four test conditions (21 and 5% oxygen with and without 3-OHB) for each cell line are shown

To investigate whether BC cells were able to use 3-OHB as a substrate for intermediate metabolism, we next determined uptake rates thereof in the panel of breast cancer cell lines. All cell lines depleted 3-OHB from the culture medium but the magnitude of depletion differed substantially between them (Fig. 3a). Interestingly, we detected variable levels of mRNA expression for key enzymes of ketolysis, namely 3-hydroxybutyrate dehydrogenase 1 (BDH1), succinyl-CoA transferase (SCOT), and acetyl-CoA-acetyltransferase (ACAT), in the seven human breast cancer cell lines (Fig. 3b). In BT474 cells, which showed a relevant consumption of 3-OHB (Fig. 3a), we detected high levels of mRNA for all three key enzymes. SCOT and ACAT, but not BDH1, were overexpressed in HBL100 cells, which showed reduced 3-OHB consumption compared to BT474 cells. All other cell lines revealed low levels of mRNA expression for ketolytic enzymes with a moderate ACAT mRNA expression in MDA-MB 231 and T47D cells. However, changes in mRNA expression levels of ketolytic enzymes in response to 3-OHB and reduced oxygen conditions varied between the tested BC cell lines. We observed an overall decrease in expression of all three enzymes in MCF7 and MDA-MB 231 cells with further decrease (MCF7) or a moderate increase of BDH1 and ACAT in the presence of 3-OHB. All enzymes were downregulated in HBL100 and MCF7 cells, but and only BDH1 and ACAT in MDA-MB 231 cells in response to low oxygen (5% oxygen). This regulation was not observed in BT20, BT474, MDA-MB 468, and T47D. In summary, the data show no strong correlation between mRNA overexpression and consumption of 3-OHB or oxygen concentration.

Moreover, the extent of 3-OHB uptake was not linked with the extent of the Warburg effect in each cell line as shown in Fig. 2a. There was also no significant difference in the concentration of 3-OHB remaining in the medium after incubation with cells at 5 or 21% oxygen (Fig. 3a). By Western blot analysis (Fig. 3c), we showed that MCT2, the key transporter for 3-OHB into cells [27], was expressed highly by all seven cell lines. In particular, MCT2 expression was higher at mild hypoxia in the majority of cell lines. Therefore, the transport of 3-OHB across the plasma membrane seems not to be a limiting factor for 3-OHB consumption by BC cells. GLUT1 as a key transporter for glucose into tumor cells was detected in all cell lines, and its expression was not influenced by 3-OHB (Fig. 3c). Together, our results demonstrate that 3-OHB does not significantly change the expression of ketolytic enzymes or of the transporter molecules MCT2 and GLUT1. Nevertheless, BC cell lines show marked differences in their ability to deplete 3-OHB from the medium indicating that mRNA expression patterns of ketolytic enzymes were not associated with consumption rate of 3-OHB.

### Short-term and long-term cell proliferation of breast cancer cells is not affected by incubation with 3-OHB

**Fig. 4:**
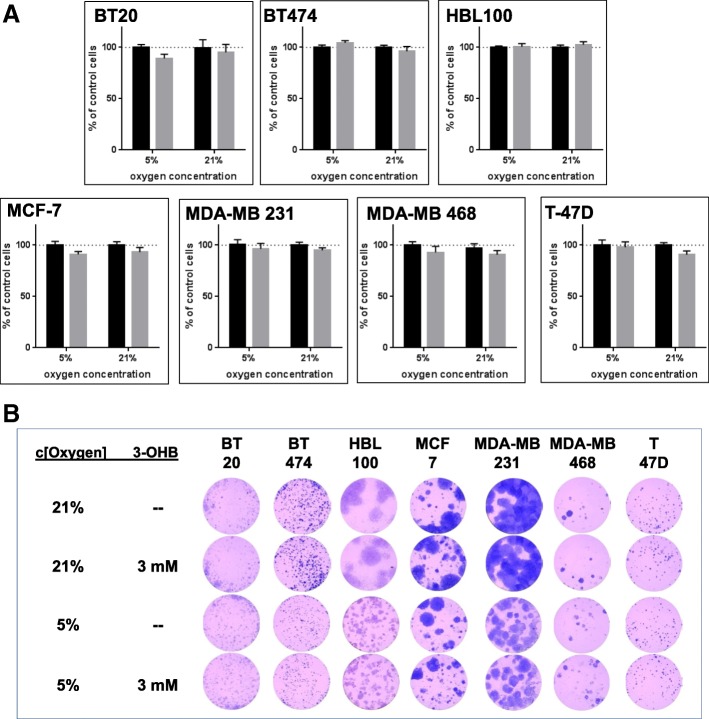
**a** The graphs show the proliferation rate (BrdU; in % of control cells) of the different BC cell lines cultured in medium containing 3 mM 3-OHB (gray column) compared to control without 3-OHB (black column) at 5 or 21% oxygen concentration after 5 days of culture (differences are not statistically significant). The columns summarize mean ± SEM of data of four independent experiments with three replicate wells per experiment for each cell line. **b** The figure shows representative results (one out of eight replicates for each cell line) of the colony formation assay for the tested BC cell lines after 14 days of culture. The cell lines show no significant alteration in number and size of colonies upon addition of 3-OHB. BT474, HBL-100, and MDA-MB 231 showed an overall reduced colony size at 5% oxygen concentration compared to 21% oxygen concentration

To analyze whether 3-OHB can affect cancer cell proliferation independent of their metabolic phenotype, we incubated the BC cell lines with 5 mM glucose, with and without addition of 3 mM 3-OHB in the presence of 5 or 21% oxygen, respectively. Here, we found a slight reduction (< 10%) in short-term (5 days) proliferation following 3-OHB treatment in BT20, MCF-7, MDA-MB 231, MDA-MB 468, and T47D cells at either oxygen concentration, while BT474 and HBL100 cells were not affected (Fig. 4a). Since AcAc, the second ketone body which rises in circulation upon a ketogenic diet, was described to increase proliferation in BRAF V600E melanoma cells [73], we performed cell proliferation assays in the presence of this metabolite over 5 days in parallel to the 3-OHB experiments. As shown in the Additional file 1, there was no significant effect of AcAc on the proliferation rate of the BC cell lines tested. A slight increase in proliferation of BT20 cells at 5% oxygen concentration did not reach statistical significance. Since AcAc was used as lithium salt, control experiments with LiCl at corresponding Li concentration were performed, but did not differ from the proliferation rates seen with Li-free cell culture medium (not shown).

To test the effect of 3 mM 3-OHB on long-term proliferation, we performed a colony formation assay for at least 14 days of culture. Similar to the results of the short-term proliferation assay, we found no significant differences in number and size of cell colonies between cultures treated with 3-OHB and control cultures. Of note, oxygen concentration influenced size and number of cell colonies of BT474, HBL100, and MDA-MB 231 cells (Fig. 4b).

### 3-OHB incubation does not influence the response of BC cells to chemotherapy or ionizing radiation

**Fig. 5:**
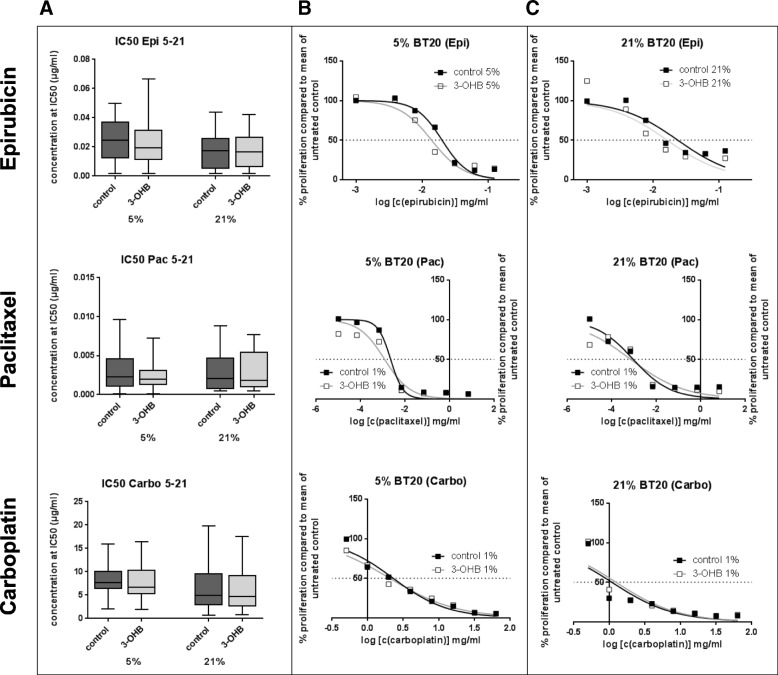
**a** The column-graphs show the cumulative IC50 of epirubicin, paclitaxel, and carboplatin in control cells (dark gray box) and cells cultured with 3 mM 3-OHB (light gray box). Per cell line, three to four each independent dose-response experiments with six replicate wells per experiment were calculated. **b** Representative dose-response curves obtained for BT-20 cells at 5% oxygen in chemotherapy sensitivity testing with the chemotherapeutic drugs (epirubicin, paclitaxel, carboplatin) which was used for the calculation of the IC50 (dashed line) (black box = control, white box = 3-OHB). **c** Same as **b** but for 21% oxygen. Curves summarize four independent experiments with six replicate wells per experiment

We next addressed the question whether exposure to 3-OHB may affect the sensitivity of BC cells to different treatment modalities. We therefore exposed the BC cell lines to epirubicin, paclitaxel, or carboplatin, three chemotherapeutic agents commonly used in breast cancer treatment. Here, we did not find any significant influence of 3-OHB on the effect of these drugs on cancer cell viability at either 5 or 21% oxygen (Fig. 5a and Additional file 2). The cumulative IC50 of paclitaxel was 4.0 ± 1.1 ng/ml (mean ± SEM) (control) versus 2.7 ± 0.8 ng/ml (3 mM 3-OHB) at 5% oxygen and 3.7 ± 1.3 ng/ml (control) versus 3.8 ± 1.2 ng/ml (3 mM 3-OHB) at 21% oxygen. The mean cumulative IC50 of epirubicin was 28.3 ± 6.1 ng/ml in control cells versus 28.5 ± 7.8 ng/ml (3 mM 3-OHB) at 5% oxygen concentration and 18.9 ± 3.6 ng/ml (control) versus 18.0 ± 2.7 ng/ml (3 mM 3-OHB) at 21% oxygen concentration. In the case of carboplatin, the mean cumulative IC50 was 6.6 ± 1.4 μg/ml (control) versus 6.0 ± 1.4 μg/ml (3-OHB) at 5% oxygen and 5.0 ± 2.4 μg/ml (control) versus 4.8 ± 2.3 μg/ml (3-OHB) at 21% oxygen concentration. Representative results of the sensitivity tests are shown in Fig. 5b, c.

**Fig. 6:**
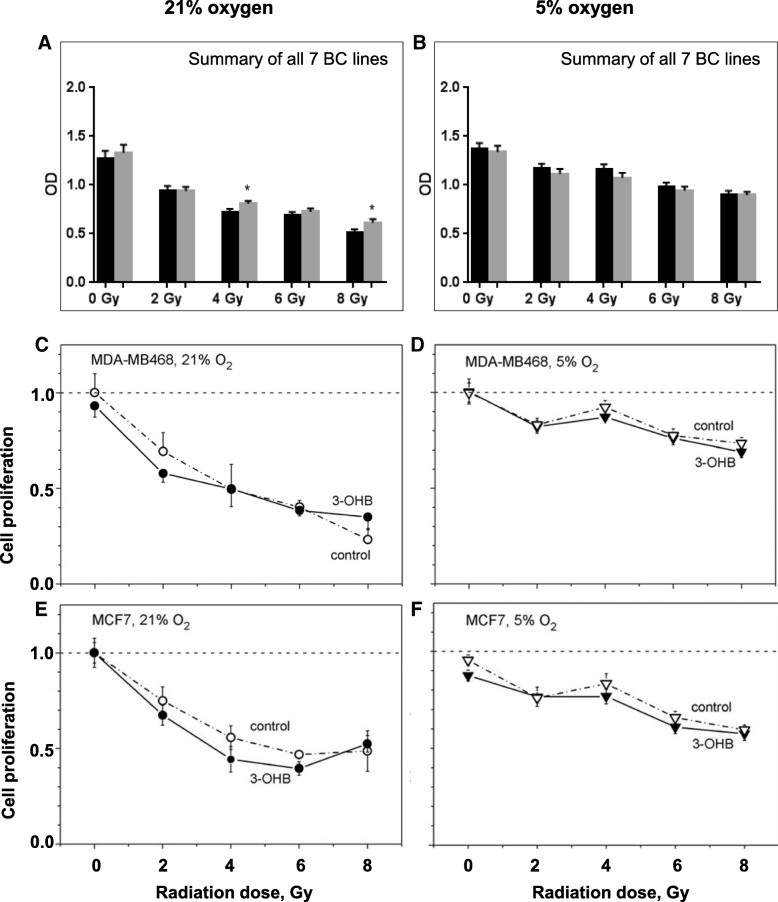
Cell proliferation after irradiation measured by BrdU, summarized for all BC cell lines at 21% (**a**) and 5% (**b**) oxygen concentration (black column = control; gray column = 3 mM 3-OHB). No significant influence of 3-OHB was seen. Columns represent mean ± SEM of three independent experiments with six replicate wells per experiment. Two representative dose-response curves for MCF7 and MDA-MB 468 are shown (**c**–**f**). MDA-MB 468 cells were sensitive to radiation (**c**, **d**), while MCF7 cells were relatively insensitive even to high doses (**e**, **f**). Open and filled symbols represent mean (± SD) of S-phase cell counts in 3-OHB-untreated control and 3-OHB-treated cells, respectively. The data were normalized to the corresponding values of non-irradiated cells at 21 or 5% oxygen, respectively

Further, we found that 3-OHB did not significantly sensitize BC cells to radiation at either 21 or 5% oxygen. Hypoxia per se, however, showed a tendency to confer a higher radio-resistance to the tumor cells (Fig. 6a + b) consistent with the known action of oxygen as a radiosensitizer. Two representative examples of dose-response curves are shown for the highest responders to radiation at both oxygen conditions in Fig. 6c–f. In analyzing the cell lines individually, however, a tendency to confer a higher radiosensitivity is seen for those tumor cells grown in 3-OHB at 5% oxygen in all but the MDA-MB 321 cell lines (Additional file 3).

## Discussion

In this study, we have shown that beta-hydroxybutyrate (3-OHB), the main ketone body found in the circulation after fasting or ketogenic diets (KDs), was able to change the energetic phenotype of BT20 breast cancer cells when applied at physiological concentrations of 3 mM. However, this effect of 3-OHB on energy metabolism was not observed in any of the other BC cell lines investigated herein. Moreover, 3-OHB did not interfere with turnover of glucose and lactate and neither significantly affected short- and long-term cancer cell proliferation, or their sensitivity to chemotherapy or ionizing radiation in any of the cell lines tested. This implies that 3-OHB at a physiological concentration of 3 mM seems to be inert in affecting energetic processes essential for proliferation or cell survival in the tested BC cell lines in vitro. In addition, we found that AcAc, the second ketone body elevated under a KD, also did not significantly influence proliferation of any BC cell line measured over 5 days. While preclinical data have shown that AcAc promotes proliferation of BRAF-V600-positive melanoma cells [73], it is interesting that in a pilot study [13] it was a patient with BRAF V600E-positive/BRAF-inhibitor-resistant melanoma who responded favorably to a KD; this highlights the limitations of translating preclinical study results to humans.

This research was performed, since a possible influence of 3-OHB on cancer growth has gained substantial interest over the past years. In particular, different regimes for KDs are currently investigated in several clinical trials to improve the outcome for cancer patients [74]. Several reports indicate a significant benefit of ketosis and especially 3-OHB on slowing cancer progression in different preclinical cancer models and patients [13, 37, 42, 44, 49, 75–77], amongst them one recent case study successfully applying a KD as part of a multimodal pro-oxidative therapy in a stage IV triple-negative breast cancer patient [78]. On the other hand, there are other publications describing a negative impact of ketosis/3-OHB on tumor cell growth in vitro and in mouse models [35, 36, 79, 80].

Therefore, we initially analyzed the energetic profile of seven different BC cell lines using substrate turnover quantification and energetic flux analysis. The basic energetic phenotypes differed somewhat from the results reported by Pelicano and coworkers [81], who found that triple-negative breast cancer (TNBC) cell lines were in general characterized by a higher extracellular acidification rate (ECAR) and lower oxygen consumption rate (OCR) compared to hormone receptor positive cell lines [81]. For the TNBC cell line BT20, we found an energetic profile that was low in OCR and ECAR and was comparable to the hormone receptor positive T47D cell line. Furthermore, the TNBC cell lines MDA-MB 231 and MDA-MB 468 showed very similar energetic profiles in our experiments and those of Pelicano and colleagues. However, in the study of Pelicano and colleagues, both MDA-MB cell lines were very similar to the energetic phenotype of BT20 cells. In contrast, in our study, the BT20 cells showed a relatively low basal OCR compared to other TNBC cell lines in our experiments. This more oxidative basal phenotype of the TNBC cell line MDA-MB 468 was recently also shown by Lanning and colleagues, even at high glucose conditions of 10 mM [82]. However, this study also described a low oxidative phenotype for the MDA-MB 231 cell line which was remarkably lower than that of MDA-MB 468, while in our investigation, both cell lines showed comparable values of OCR. It is likely that these discrepancies between metabolic profiling data can arise from small differences in the culture conditions, for example different metabolite concentrations in fetal calf serum. Nevertheless, we can conclude that under the experimental conditions used here, only BT20 cells shifted their metabolic profile upon exposure to reduced oxygen concentration. Those discrepancies between our and other metabolic profiling data could also be due to different expression profiles of the cell lines in different laboratories based on different subclones [83, 84].

Of note, our quotient of OCR/ECAR for BT20 had a range from 0.9–2.4 (not shown), which is closer to the original findings of Warburg, who found that the quotient of respiration to aerobic glycolysis was 0.3–0.9 in tumor tissue sections [8, 9]. Apart from BT20, the other BC cell lines remained relatively stable with respect to the OCR/ECAR ratio in the presence of 3-OHB.

We found that 3-OHB increases both basic OCR and maximal OCR after inhibition of ATP synthesis by oligomycin and in the presence of the uncoupling agent FCCP in BT20 cells. This indicates that 3-OHB alters mitochondrial capacity, for example, by increasing the expression/activity of respiratory complexes or by inducing mitochondrial biogenesis. In addition, glycolysis was also increased after 3-OHB exposure, indicating an overall increase in metabolic activity of these cells (as displayed in Fig. 2a). However, this phenotype was not observed in the other breast cancer cell lines used in our study. One reason for this difference could be that BT20 is the only TNBC cell line that is mutant for BRCA2 (Table 1). Indeed, a large-scale metabolomics study recently found that BT20 cells differ in their metabolic profile compared to other TNBC cells, based on unsupervised hierarchical clustering [85]. While further experimentation is required to identify the underlying mechanisms for the overall increase in metabolic activity caused by 3-OHB exposure in these cells, our data clearly show that 3-OHB does not affect the viability of BT20 cells or their response to chemotherapy or radiation, despite this metabolic effect. Notably, ECAR is not only dependent on medium acidification via lactic acid but can also be affected by the production of carbonic acid (H_2_CO_3_) as an end product of the oxidative degradation of metabolites in the tricarboxylic acid (TCA) cycle [86, 87]. Here, a very important aspect is the observation that breast cancer cell lines, and especially the TNBC cell lines, often use glutamine as a relevant nutrient to support their metabolic demands [88, 89]. Moreover, glutamine can also be converted to pyruvate and lactate by malic enzyme [90]. Since glutamine is not a limiting factor in our cell culture medium, we cannot exclude that the observed increase in ECAR is due to glutamine metabolism in the cell lines investigated. Therefore, the increase in ECAR in parallel to elevated OCR in BT20 cells is likely to correspond to a generally more “energetic phenotype” of enhanced respiration and CO_2_ or lactate-induced acidification [86, 87]. Another possible interpretation would be mitochondrial uncoupling via overexpression of uncoupling protein 2 (UCP2) that, although not evaluated in our study, has been shown to occur in BC cell lines [91]. UCP2 overexpression has a metabolic action by supporting glucose and glutamine fermentation at the expense of mitochondrial oxidation [92]. If mitochondrial uncoupling increased, then oxygen consumption would not strictly be linked to respiratory capacity.

Anyway, the shift to a more energetic phenotype seems not to correlate with the metabolism of 3-OHB as an energy source. As proven by RT-qPCR, the BT20 cells express very low levels of mRNA for BDH1, the key entry enzyme of ketolysis, and in line with this, consume only small amounts of 3-OHB. Of note, BT20 cells also displayed the highest basal rate of glucose consumption and lactic acid production of all cell lines tested and this rate was not influenced by 3-OHB at physiological concentrations. Further, the proliferation rate of BT20 cells was unaffected by 3-OHB, similar to the other six cell lines tested.

Here, we found no evidence that 3-OHB fuels the metabolism of BC cells in vitro. This is in accordance to an in vivo study, which showed that 3-OHB did not influence growth of melanoma cells either when injected intraperitoneally or when elevated by a high-fat diet [73]. However, at the same time, it is in contrast to a previous study that has shown an increased growth of breast cancer xenografts derived from MDA-MB 231 cells when mice were injected with 3-OHB intraperitoneally [36]. In our in vitro experiments, the MDA-MB 231 cell line showed no increased proliferation in the presence of 3-OHB. Moreover, no notable inhibition of proliferation by 3-OHB could be seen in our short-term (5 days) and long-term (at least 14 days) experiments, as described for other cell lines [39–41]. In this context, it should be noted that the growth inhibitory effects for 3-OHB previously reported were predominantly seen with very high and non-physiological concentrations of 3-OHB (5–40 mM), an observation we have seen in our cell lines as well (not shown). In detail, the first description of an antiproliferative effect of 3-OHB on different cancer cell lines was published by Magee et al. in 1979. The authors tested concentrations of 3-OHB between 10 and 40 mM [39]. In 2009, Skinner et al. described an effect of 3-OHB on the viability of human neuroblastoma cells. Again, the authors used very high concentrations of 3-OHB between 24 and 43 mM [41]. Interestingly, an antiproliferative effect of 3-OHB was also described for a physiological concentration of 5 mM 3-OHB in brain tumor cells, but at a very high glucose concentration of 25 mM that represents a more pathophysiological ketoacidotic situation [40]. Our intent was to investigate the effect of 3-OHB in vitro under conditions more likely to be found in cancer patients on KDs. That is why we used physiological concentration of 3-OHB (3 mM) and glucose (5 mM) [22–24]. Using cell culture conditions comparable to our experiments, Martuscelli described an antiproliferative effect of 3-OHB in glioblastoma cell lines and tumor stem cells with half maximal inhibitory concentration (IC50) of 2 mM 3-OHB in the presence of low (4 mM) and physiological (5–6 mM) glucose concentrations [37]. These contrary results described for the effect of 3-OHB on BC cells and glioma cells may reflect differences in their ability to consume 3-OHB.

Previous studies have shown variable gene expression levels of key enzymes involved in ketolytic metabolism in cancer cell lines of different entities [41, 93–97]. In line with these data, we found different mRNA expression patterns of ketolytic enzymes in BC cell lines. In BT474 and HBL100, mRNA transcripts for all ketolytic enzymes were detectable. This was associated with the highest relative consumption rate of 3-OHB by these cells. In contrast, the other BC cell lines failed to express increased levels of at least one of these key enzymes. This mRNA expression pattern was independent from the subtype of BC [59–61] (Table 1) and not influenced by 3-OHB. In accordance to the results of Antalis and colleagues [98], we found very low mRNA transcripts for ACAT1 for BDH1 in MCF-7 cells, which was linked to their inability to consume 3-OHB. Altogether, the expression of ketolytic enzymes on mRNA level seems not to be associated with the rate of 3-OHB consumption and unrelated to levels of glucose consumption and lactate production; however, we are aware, that mRNA expression did not allow to judge about enzymatic activity.

Cells take up ketone bodies by monocarboxylate transporters (MCTs), a family of proton-linked plasma membrane transporters that carry ketone bodies across biological membranes. The most important transporter for 3-OHB into cancer cells is MCT2 [25, 99–101], and previous studies describe an overexpression of MCT2 in BC cells [102]. Here, we found a strong expression of MCT2 in all seven BC cell lines, so that the absence of 3-OHB effects in the cells cannot be explained by defective MCT expression. Since the expression of GLUT1 was found to be related to poor prognosis in breast cancer [103, 104], we analyzed if its expression could be reduced by 3-OHB. However, as for MCT2, we could not detect any modulation of the GLUT1 receptor expression in dependency of 3-OHB, an observation also described in cardiomyocytes [105].

The KD is increasing in popularity, and an increasing number of cancer patients are trying the KD simultaneously with chemotherapy and radiation therapy. Recent clinical trials, e.g., NCT01419483, investigate safety and tolerance of a KD during combined chemotherapy and radiation. We analyzed the response of 3-OHB-treated BC cells to chemotherapy and radiation in vitro. No significant changes in the dose-response to three chemotherapeutical drugs most commonly used in BC treatment [106–110] were observed. Thus, the sensitizing effect of a ketogenic diet on radiochemotherapy in vivo [111] might be mediated by effects other than direct influences on cancer cells. In this context, the clinical study published by Klement and Sweeney [112] is of interest. The authors described an adequate tumor regression for a small cohort of cancer patients undergoing a KD and radiation therapy. Further, two mouse studies with glioma and lung cancer confirm the radio-sensitizing effect of a ketogenic diet [111, 113]. To date, no information is available on a possible radio-sensitizing effect of 3-OHB. Our in vitro results with 3 mM 3-OHB indicate a non-significant tendency of this ketone body to sensitize most BC cells to ionizing radiation.

## Conclusions

The intent of the study was to investigate the effect of 3-OHB on seven BC cell lines in vitro under conditions likely to be found in patients on a KD or short-term starvation. We have found strong evidence that a physiological concentration of 3 mM 3-OHB and AcAc did not impact cell proliferation and the response to standard BC chemotherapy and ionizing radiation is not changed by 3-OHB. These findings were independent from the diverse genetic background of the cell lines and differences in mRNA expression of ketolytic enzymes and 3-OHB uptake. Taken together, we found that 3-OHB at physiological concentrations has no major impact on BC cell proliferative behavior and the metabolic activity in vitro and especially does not fuel tumor cell growth. These results support clinical observations that physiologically increased 3-OHB serum concentrations induced either by a ketogenic diet or by short-term starvation do neither support nor inhibit breast cancer cell proliferation. Thus, a ketogenic diet should be safe for breast cancer patients as already described for patients with diverse cancer types (for review, see [114]).

## Additional files

Additional file 1: The graphs show the proliferation rate (BrdU; in % of control cells) for the seven different breast cancer cell lines cultured in medium containing 1.5 mM AcAc (white column) compared to control without AcAc (black column) at 5% or 21% oxygen concentration after 5 days of culture (differences are not statistically significant). The columns represent mean ± SEM of data of 4 independent experiments with 3 replicate wells per experiment for each cell line. (PPTX 104 kb)

Additional file 2: Graphs present the IC50 with the 95% confidence intervals for the seven tested cell lines obtained for the three cytostatic drugs epirubicin, paclitaxel and carboplatin comparing the IC50 obtained for cells cultured with 3 mM 3-OHB (gray blots) with the control cells grown in medium free of 3-OHB (black boxes). Each blot represents 3–4 independent dose-response experiments with 6 replicate wells per experiment. None of the differences are statistically significant; however a strong tendency to a reduction in IC50 of paclitaxel is seen for T47D grown in 3-OHB medium compared to the control. (PPTX 104 kb)

Additional file 3: Columns represent mean ± SEM of cell proliferation after irradiation shown for the seven cell lines at 21% and 5% oxygen concentration (gray column = with 3-OHB; black column without 3-OHB) (summarized in Fig. 6). The BT20, BT474 and T47D cell lines cultured in the presence of 3-OHB showed a trend towards increased radio-resistance at 21% oxygen (with some significant results at single doses). In contrast, in MCF-7 and MDA-MB 468, 3-OHB cultured cells showed a trend towards impaired cell proliferation following radiation at the same oxygen concentration. At 5% oxygen concentration, 3-OHB seemed to have a sensitizing effect to radiation in some cell lines. Columns represent mean ± SEM of 3 independent experiments with 6 replicate wells per experiment. *< 0.05, **p < 0.01, ***p < 0.001. (PPTX 152 kb)

## Abbreviations

3-OHB: Beta-hydroxybutyrate
AcAc: Acetoacetate
ACAT: *Homo sapiens* acetyl-CoA-acetyltransferase
BDH: 3-Hydroxybutyrate dehydrogenase
BrdU: 5-Bromo-2′-deoxyuridine
CLS: Cell Lines Service GmbH
DMEM: Dulbecco’s modified Eagle’s medium
ECAR: Extracellular acidification rate
FCS: Fetal calf serum
GLUT1: Glucose transporter 1
KD: Ketogenic diet
MCT: Monocarboxylic acid transporter
mpH: Milli pH
MW: Molecular weight
OCR: Oxygen consumption rate
OD: Optical density
OXPHOS: Oxidative phosphorylation
PPIA: Peptidylprolyl isomerase A
SCOT: Succinyl-CoA transferase
SEM: Standard error of mean
TDC: Test drug concentration
TNBC: Triple-negative breast cancer
