# Supplementary figures and images for: Beta-hydroxybutyrate (3-OHB) can influence the energetic phenotype of breast cancer cells, but does not impact their proliferation and the response to chemotherapy or radiation
Source: Cancer Metab. 2018 Jun 11;6:8. doi: 10.1186/s40170-018-0180-9 (PMC5996481; doi:10.1186/s40170-018-0180-9)

## Slide 1
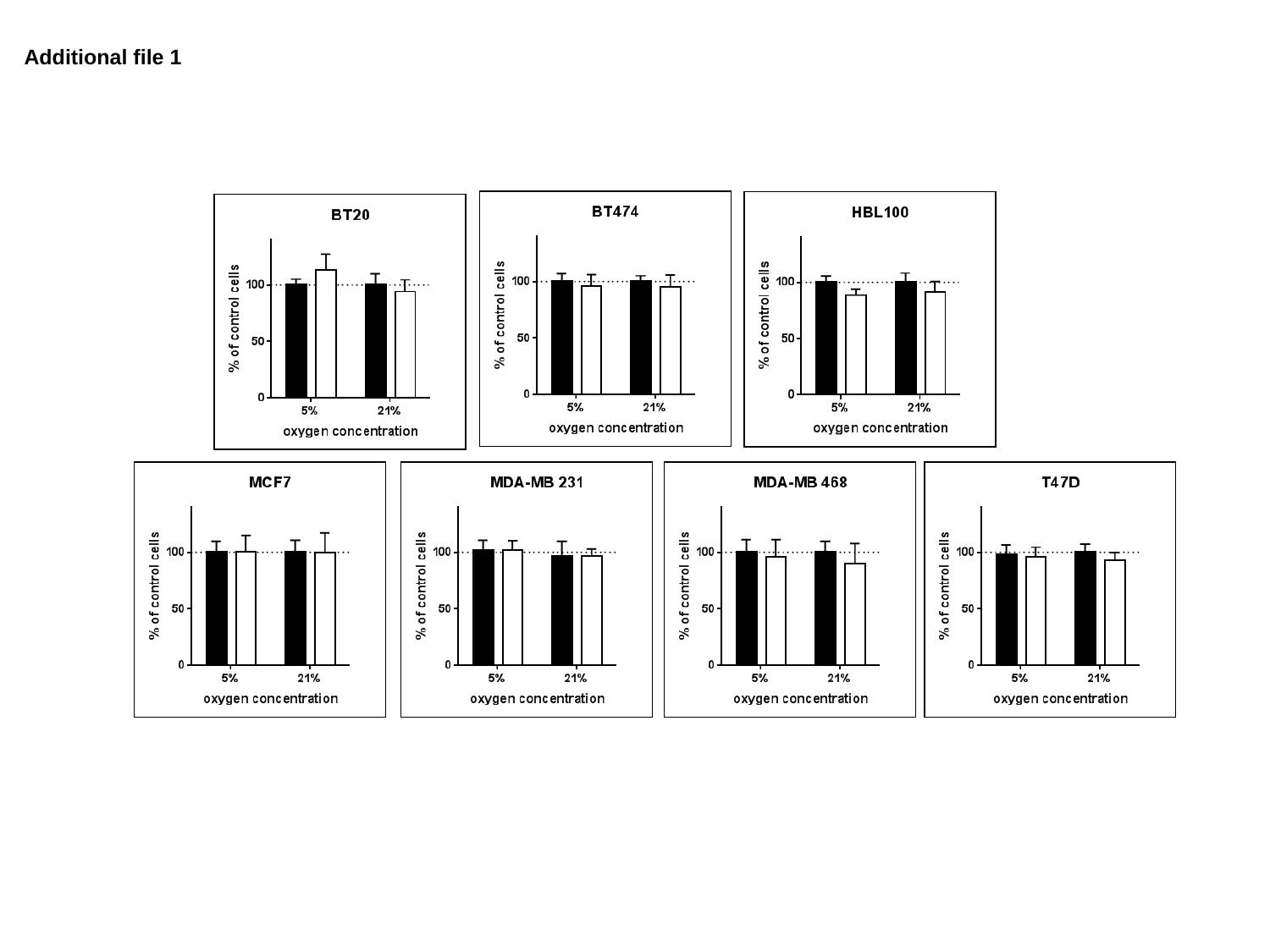

Additional file 1

Supplement: Supplementary file 1 — The graphs show the proliferation rate (BrdU; in % of control cells) for the seven different breast cancer cell lines cultured in medium containing 1.5 mM AcAc (white column) compared to control without AcAc (black column) at 5% or 21% oxygen concentration after 5 days of culture (differences are not statistically significant). The columns represent mean ± SEM of data of 4 independent experiments with 3 replicate wells per experiment for each cell line. (PPTX 104 kb) [file 40170_2018_180_MOESM1_ESM.pptx]

## Slide 1
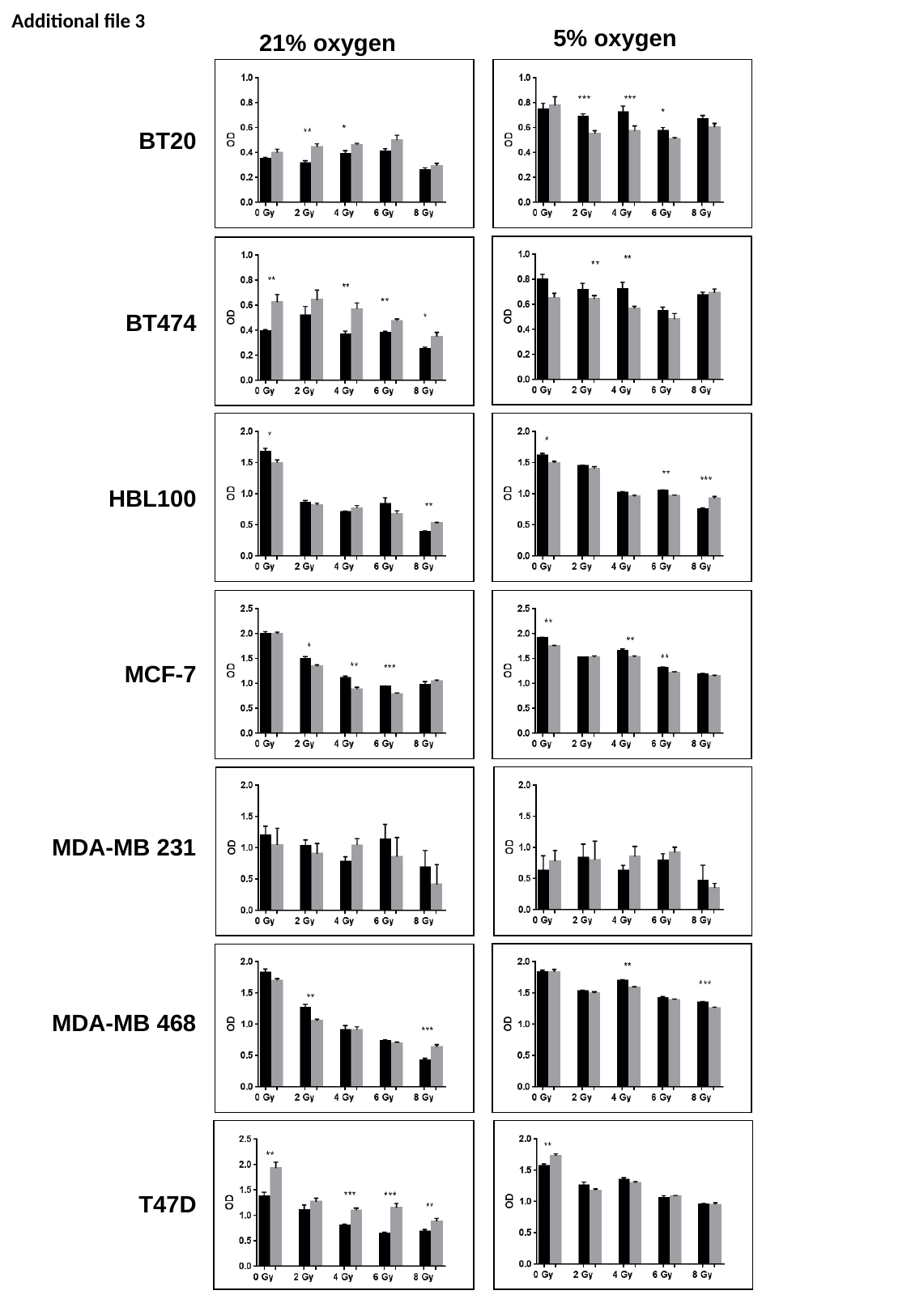

Additional file 3
5% oxygen
21% oxygen
BT20
BT474
HBL100
MCF-7
MDA-MB 231
MDA-MB 468
T47D

Supplement: Supplementary file 3 — Columns represent mean ± SEM of cell proliferation after irradiation shown for the seven cell lines at 21% and 5% oxygen concentration (gray column = with 3-OHB; black column without 3-OHB) (summarized in Fig. 6). The BT20, BT474 and T47D cell lines cultured in the presence of 3-OHB showed a trend towards increased radio-resistance at 21% oxygen (with some significant results at single doses). In contrast, in MCF-7 and MDA-MB 468, 3-OHB cultured cells showed a trend towards impaired cell proliferation following radiation at the same oxygen concentration. At 5% oxygen concentration, 3-OHB seemed to have a sensitizing effect to radiation in some cell lines. Columns represent mean ± SEM of 3 independent experiments with 6 replicate wells per experiment. *< 0.05, **p < 0.01, ***p < 0.001. (PPTX 152 kb) [file 40170_2018_180_MOESM3_ESM.pptx]
