# Supplementary material for: Beta-hydroxybutyrate (3-OHB) can influence the energetic phenotype of breast cancer cells, but does not impact their proliferation and the response to chemotherapy or radiation
Source: Cancer Metab. 2018 Jun 11;6:8. doi: 10.1186/s40170-018-0180-9 (PMC5996481; doi:10.1186/s40170-018-0180-9)
Supplement: Supplementary file 2 — Graphs present the IC50 with the 95% confidence intervals for the seven tested cell lines obtained for the three cytostatic drugs epirubicin, paclitaxel and carboplatin comparing the IC50 obtained for cells cultured with 3 mM 3-OHB (gray blots) with the control cells grown in medium free of 3-OHB (black boxes). Each blot represents 3–4 independent dose-response experiments with 6 replicate wells per experiment. None of the differences are statistically significant; however a strong tendency to a reduction in IC50 of paclitaxel is seen for T47D grown in 3-OHB medium compared to the control. (PPTX 104 kb) [file 40170_2018_180_MOESM2_ESM.pptx]

## Slide 1
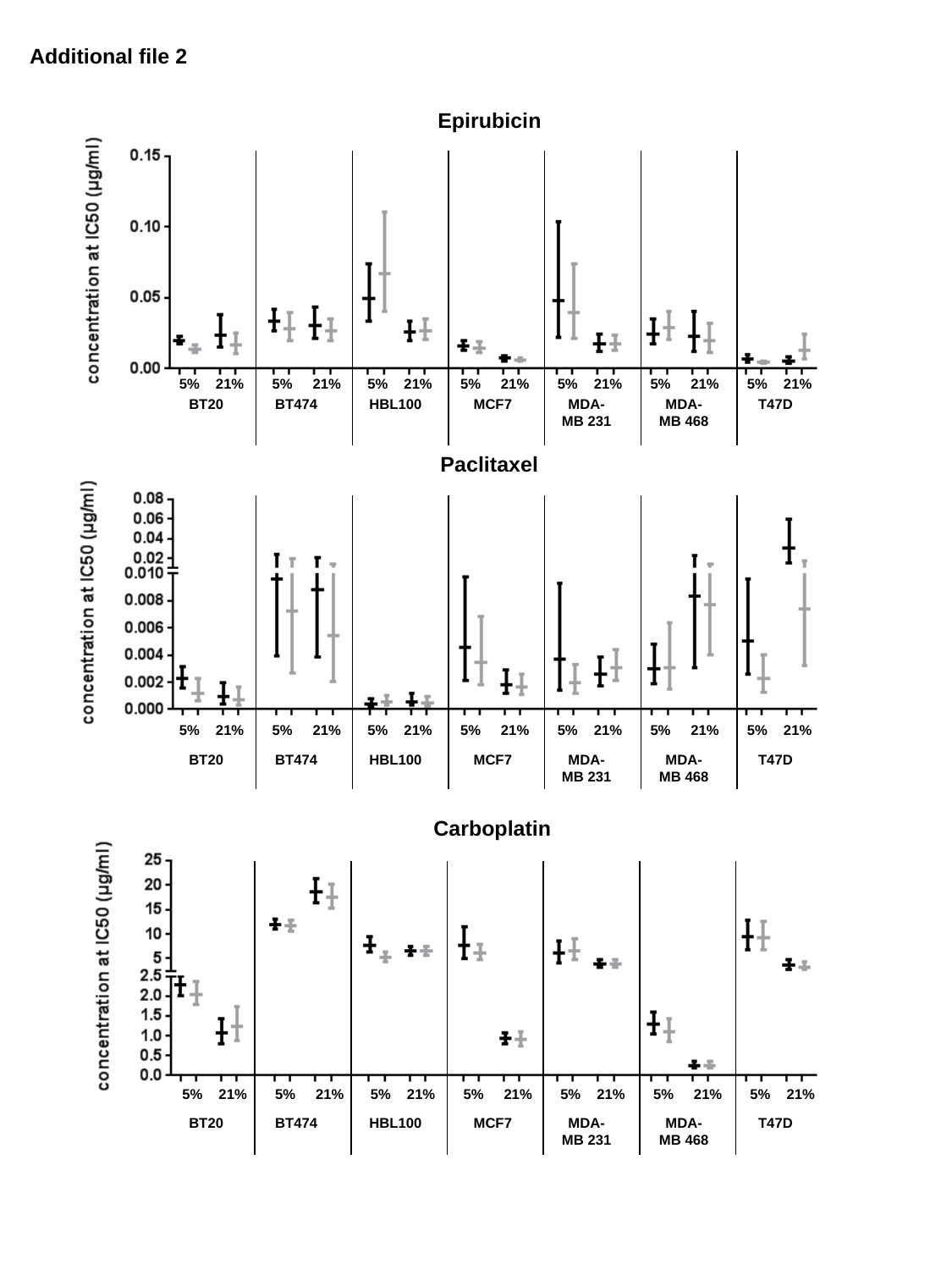

Additional file 2
Epirubicin
5% 21% 5% 21%
5% 21% 5% 21%
5% 21% 5% 21%
5% 21%
BT20
BT474
HBL100
MCF7
MDA-
MB 231
MDA-
MB 468
T47D
Paclitaxel
5% 21% 5% 21%
5% 21% 5% 21%
5% 21% 5% 21%
5% 21%
BT20
BT474
HBL100
MCF7
MDA-
MB 231
MDA-
MB 468
T47D
Carboplatin
5% 21% 5% 21%
5% 21% 5% 21%
5% 21% 5% 21%
5% 21%
BT20
BT474
HBL100
MCF7
MDA-
MB 231
MDA-
MB 468
T47D
